# Dose-Painted Intensity Modulated Radiation Therapy Improves Local Control for Locally Advanced Pancreas Cancer

**DOI:** 10.5402/2012/572342

**Published:** 2012-10-18

**Authors:** Ahmet Tunceroglu, Joo Han Park, Sairam Balasubramanian, Matthew Poppe, Christopher J. Anker, Elizabeth Poplin, Rebecca A. Moss, Ning J. Yue, Darren Carpizo, Christopher J. Gannon, Bruce G. Haffty, Salma K. Jabbour

**Affiliations:** ^1^Department of Radiation Oncology, The Cancer Institute of New Jersey, Robert Wood Johnson Medical School, University of Medicine and Dentistry of New Jersey, G2N45, 195 Little Albany Street, New Brunswick, NJ 08903, USA; ^2^Department of Radiation Oncology, University of Utah School of Medicine, Huntsman Cancer Hospital, 1950 Circle of Hope, Room 1570, Salt Lake City, UT 84112, USA; ^3^Division of Medical Oncology, Department of Medicine, The Cancer Institute of New Jersey, Robert Wood Johnson Medical School, University of Medicine and Dentistry of New Jersey, 195 Little Albany Street, New Brunswick, NJ 08903, USA; ^4^Department of Surgery, The Cancer Institute of New Jersey, Robert Wood Johnson Medical School, University of Medicine and Dentistry of New Jersey, 195 Little Albany Street, New Brunswick, NJ 08903, USA; ^5^Advanced Surgical Associates of New Jersey, Two Capital Way, Suite 356, Pennington, NJ 08534, USA

## Abstract

*Background*. To evaluate the outcomes, adverse events, and therapeutic role of Dose-Painted Intensity-Modulated Radiation Therapy (DP-IMRT) for locally advanced pancreas cancer (LAPC). *Methods*. Patients with LAPC were treated with induction chemotherapy (*n* = 25) and those without metastasis (*n* = 20) received DP-IMRT consisting of 45 Gy to Planning Treatment Volume 1 (PTV1) including regional lymph nodes with a concomitant boost to the PTV2 (gross tumor volume + 0.5 cm) to either 50.4 Gy (*n* = 9) or 54 Gy (*n* = 11) in 25 fractions. DP-IMRT cases were compared to three-dimensional conformal radiation therapy (3D-CRT) plans to assess the potential relationship of radiation dose to adverse events. Kaplan-Meier and Cox regression analyses were used to calculate survival probabilities. The Fisher exact test and *t*-test were utilized to investigate potential prognostic factors of toxicity and survival. *Results*. Median overall and progression-free survivals were 11.6 and 5.9 months, respectively. Local control was 90%. Post-RT CA-19-9 levels following RT were predictive of survival (*P* = 0.02). Grade 2 and ≥grade 3 GI toxicity were 60% and 20%, respectively. In comparison to 3D-CRT, DP-IMRT plans demonstrated significantly lower V45 values of small bowel (*P* = 0.0002), stomach (*P* = 0.007), and mean liver doses (*P* = 0.001). *Conclusions*. Dose-escalated DP-IMRT offers improved local control in patients treated with induction chemotherapy for LAPC. Radiation-related morbidity appears reduced with DP-IMRT compared to 3D-CRT techniques, likely due to reduction in RT doses to organs at risk.

## 1. Introduction

Pancreatic cancer is currently the fourth leading cause of cancer-related deaths in the United States [1]. There were an estimated 37,680 new cases in 2008 and that approximation has now risen to 43,920 for 2012 [1, 2]. Pancreatic cancer has a very poor overall survival, due in part to the fact that less than 15% of patients have resectable disease at diagnosis [3]. Furthermore, an estimated 15–50% of patients who were initially thought to have resectable cancer will be found at the time of surgery to have unresectable or metastatic disease [3], suggesting an aggressive biology of this cancer. Various modalities have been developed for the treatment of locally advanced pancreatic cancer (LAPC), including chemotherapy alone, chemotherapy followed by chemoradiotherapy, stereotactic body radiotherapy (SBRT), and combination regimens of chemoradiotherapy and SBRT [4–6]. Despite the judicious application of such treatment modalities, LAPC continues to have an extremely poor prognosis with median survival rates range between 7.9 and 11 months. Although death is from metastatic disease in 70% of patients, the remaining 30% succumb to poor local control [7–9].

 A common limitation in pancreatic cancer radiation therapy has been the toxicity thought to result from irradiation of nearby organs, most prominently the small bowel and/or stomach. Published studies of various chemoradiotherapy regimens have demonstrated a wide variety of adverse events ranging from occasional nausea to grade 4 vomiting. Evidence suggests that intensity-modulated radiation therapy (IMRT) may provide superior limitation of radiation toxicity, likely due to a dose reduction to organs at risk (OARs). Indeed, recent studies have shown successful OAR dose reduction for other upper abdominal cancers using IMRT as compared to three-dimensional conformal radiotherapy (3D-CRT). For example, in the postoperative treatment of gastric and gallbladder cancer, statistically significant reduced doses and toxicity to adjacent liver and kidneys have been observed [10, 11].

Whether or not the success that has been seen in the treatment of other malignancies with IMRT is transferable to the management of pancreatic cancer is a subject of ongoing research. For this study, we hypothesized that the incorporation of Dose-Painted IMRT (DP-IMRT) in the management of locally advanced pancreas cancer (LAPC) would reduce side effects due to a decrease in doses to the small bowel, stomach, and liver. DP-IMRT allows for a higher dose per fraction to be delivered to the primary pancreas tumor, while simultaneously treating regional lymph nodes at standard fractionation to doses required for eradication of microscopic disease. Therefore, DP-IMRT may mitigate adverse effects through improved OAR sparing while hypofractionation to the primary tumor may improve clinical response. Here we report on the use of IMRT in the treatment of LAPC with a focus on local control rates, survival, and toxicity. Findings are compared to recently published studies utilizing 3D-CRT in combination with various chemotherapeutic agents [9, 12].

## 2. Methods and Materials

### 2.1. Patient Demographics and Treatment Regimen

All patients (*n* = 26) had a diagnosis of locally advanced pancreatic adenocarcinoma, of whom half were T4 and node positive (38%). Median patient age was 66 years (range, 40–87). Patients receiving DP-IMRT (*n* = 20) were evaluated by surgical, medical, and radiation oncologists and were all deemed to be borderline resectable (*n* = 4) or unresectable (*n* = 16). Almost all patients (96%) received induction chemotherapy followed by computed tomography (CT) restaging. If there was no evidence of metastatic disease, patients were then treated with DP-IMRT concurrent with chemotherapy. Four to six weeks following the completion of chemoradiation therapy, patients received two additional cycles of chemotherapy or proceeded with surgery if candidacy was established. When given in the induction setting, full-dose gemcitabine was used (1000 mg/m^2^), whereas 400 mg/m^2^ was used concurrently with RT.  Table 1 shows details regarding patient demographics and their treatment.

### 2.2. DP-IMRT Planning

Tissue constraints were as previously published [13]. Liver V35 (i.e., the % volume of the liver receiving 35 Gy or more) was limited to ≤33%, and V45 for the stomach was limited to ≤10% with V54 < 3%. The small bowel V45 was kept to ≤10% with V54 < 3%. The entire spinal cord was limited to 45 Gy, and the kidney dose was limited so that ≤50% of the total kidney volume would receive more than 18 Gy.

Gross Tumor Volume (GTV) was determined based on CT findings, and margins of 1 cm were added to establish the Clinical Target Volume (CTV). Planning Treatment Volume 1 (PTV1), which included regional pancreaticoduodenal, celiac, para-aortic, and portahepatic lymph nodes, received a total dose of 45 Gy. Dose escalation was delivered to PTV2 (GTV + 0.5 cm) using either 50.4 Gy (*n* = 9) or 54 Gy (*n* = 11) in 25 fractions. Radiation was administered daily Monday through Friday with the goal of delivering ≥45 Gy to ≥95% of PTV1 (*n* = 19), although ≥90% was acceptable (*n* = 1). Upper dose constraints were set to be equal to 55.4 Gy and 59.4 Gy to ≤5% of PTV2 for the 50.4 and 54 Gy dose escalations, respectively. Planning incorporated an ITV and respiratory motion was managed with respiratory gating or abdominal compression. Daily image guidance consisting of orthogonal kV images and cone beam CT was utilized during radiation therapy.

Ten randomly selected DP-IMRT plans for patient treatment were subsequently replanned with 3D-CRT to evaluate the radiation doses to organs at risk (OARs) and the potential relationship of this exposure to the observed adverse events. Of these 10 patients, 6 were treated with dose escalation to 54 Gy. For comparison 3D-CRT plans, both coplanar and noncoplanar plans were designed as previously described [13]. For DP-IMRT a coplanar beam arrangement was used.

### 2.3. Data Collection and Analysis

During the course of their treatment and at routine follow-up visits, patients were asked a standard questionnaire pertaining to commonly occurring toxicities consisting of nausea, vomiting, diarrhea, abdominal pain, anorexia, fatigue, and weight loss. Responses were categorized according to Common Terminology Criteria for Adverse Events (CTCAE) version 4.0 guidelines. Morbidity was assessed prior to, during, and following treatment. Response Evaluation Criteria In Solid Tumors (RECIST) criteria version 1.1 were used to define progression of disease. Locoregional cancer recurrence was defined as recurrence within the tumor bed and regional lymph nodes. Metastatic recurrence was defined as distant failure outside the radiation field. Preradiation CA 19-9 levels were obtained 4–6 weeks prior to RT during the last cycle of chemotherapy and postradiation CA 19-9 levels were collected at a median of 3 weeks (range 0.5–18) following the completion of RT.

### 2.4. Statistical Methods

The Fisher exact test and *t*-test were used to identify statistically significant prognostic factors of adverse events. Kaplan-Meier and Cox regression analyses were performed to evaluate median overall (OS) and progression-free (PFS) survivals as well as predictors of survival. Statistically significant differences in the occurrence of adverse events between the group of patients receiving dose escalation to 50.4 Gy and those receiving 54.0 Gy were determined using the Fisher exact test. The Fisher exact test was also used to investigate potential associations between GTV and adverse events by treating GTV as a dichotomous variable (≥ or <100 and 200 cm^3^). Prognostic significance of patient characteristics for survival was analyzed using Cox regression analyses. Comparison of radiation exposure to OARs between IMRT and 3D-CRT was made with use of the *t*-test. SPSS Statistics version 20 was used for statistical analysis.

## 3. Results

### 3.1. Clinical Outcomes

**Figure 1:**
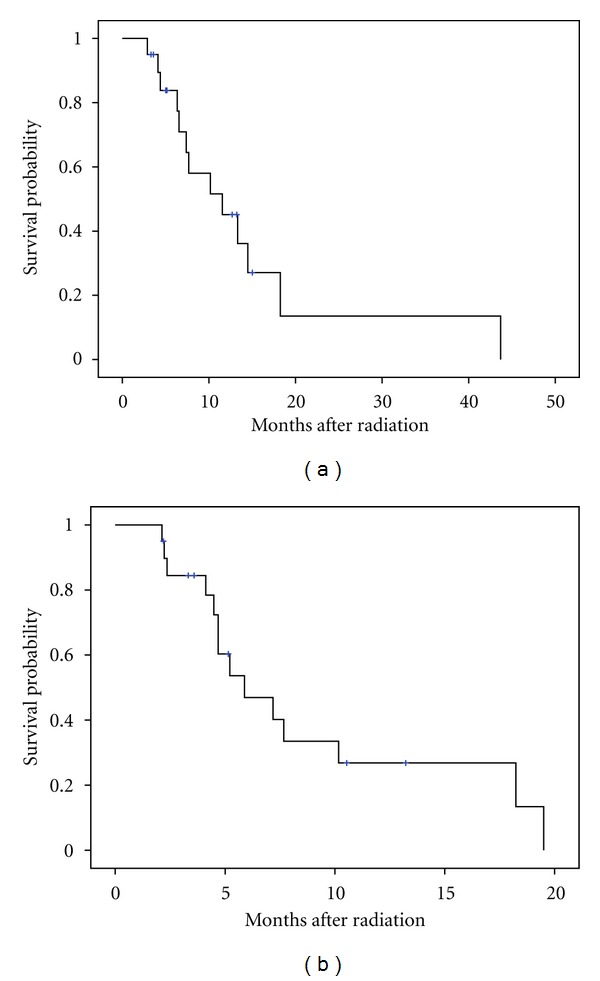
Kaplan-Meier curves for patients without evidence of metastatic disease on CT imaging (*n* = 20) who proceeded to receive radiation. (a) Median and 1-year overall survival in this study were 11.6 months and 55%, respectively. (b) Median and 1-year progression free survival were 5.9 months and 15%, respectively.

Of the original 26 patients in our cohort, 77% (*n* = 20) did not show evidence of metastatic disease on CT restaging and proceeded to receive DP-IMRT concurrently with chemotherapy. The median follow-up time for living patients was 8.9 months with median OS of 11.6 months and a PFS of 5.9 months (Figure 1). The 1-year OS and locoregional control (LRC) rates among patients receiving DP-IMRT were 55% and 90%, respectively. Seventeen patients had stable disease, with one who had a partial response and another with pathologic complete response. The response of 1 patient who was lost to follow-up could not be determined. Cause of death at 1 year was related to metastatic disease in 35% and from locoregional progression in 5%.

Among the adverse events noted in our study, the most commonly reported were diarrhea, nausea, vomiting, anorexia, abdominal pain, fatigue, and weight loss. The toxicities noted were mostly grade 1 or 2 with the most common being grade 1 nausea (45%) and grade 1 fatigue (45%). One patient exhibited grade 3 nausea and anorexia, and one patient experienced grade 3 anorexia only. Two patients developed grade 3 gastrointestinal (GI) bleeding, one of which was a result of local failure, and 1 patient developed grade 3 bile duct stenosis. There were no grade 4 adverse events noted (Table 2). Grade 1 and 2 anemia were also noted, each exhibited by 45% of patients, with only one patient developing grade 3 anemia (Table 2). There was no significant difference in the occurrence of diarrhea (*P* = 1.00), nausea (*P* = 0.7), vomiting (*P* = 0.7), anorexia (*P* = 1.00), abdominal pain (*P* = 0.6), fatigue (*P* = 0.5), or weight loss (*P* = 0.4) between the patients who received 50.4 Gy and those who received 54.0 Gy. Four patients subsequently proceeded to surgery and one died of postoperative complications. 

### 3.2. Predictors and Prognostic Factors of Toxicity and OS

**Figure 2:**
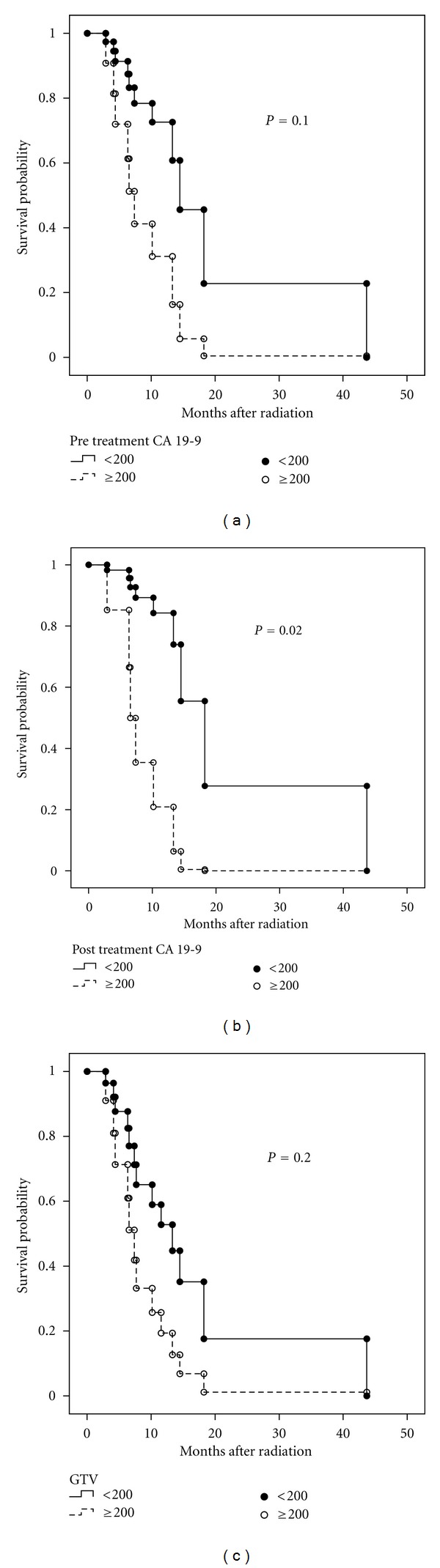
Kaplan-Meier curves for patients receiving Dose-Painted Intensity Modulated Radiation Therapy (DP-IMRT) for (a) pretreatment CA 19-9 levels, (b) posttreatment CA 19-9 levels, and (c) GTV.

Univariate analysis for prognostic factors of OS yielded a statistically significant association with postradiation CA 19-9 levels but not preradiation CA 19-9 levels (*P* = 0.02 and *P* = 0.1, resp.) (Figures 2(a) and 2(b)). GTVs of ≥100 cm^3^ or 200 cm^3^ did not have prognostic significance with regard to OS (*P* = 0.9 and *P* = 0.2, resp.) (Figure 2(c)).

Given that the common gastrointestinal (GI) toxicities of RT are thought to be due to irradiation of crucial nearby OARs, it is reasonable to hypothesize that the volume of tumor targeted for irradiation would correlate strongly and directly with the severity of adverse events. Among the adverse events noted in our study, GTVs > 200 cm^3^ correlated with the degree of abdominal pain (*P* = 0.03) and nausea (*P* = 0.03).

### 3.3. Radiation Dose to OARs

**Figure 3:**
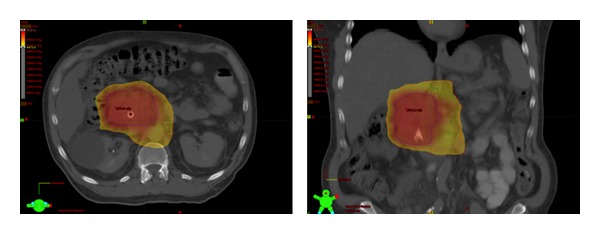
Axial and coronal images of representative DP-IMRT plans depicting dose wash of ≥42.8 Gy to PTV1 (yellow) and dose escalation of 53.9–57.3 Gy to PTV2 (red). DP-IMRT: Dose-Painted Intensity Modulated Radiation Therapy PTV: Planning Treatment Volume.

Comparing radiation exposure of organs at risk between coplanar 3D-CRT and noncoplanar 3D-CRT, treatment with noncoplanar 3D-CRT resulted in significantly lower V12 of the right kidney (*P* = 0.03), V18 of the left kidney (*P* = 0.04), V45 of the small bowel (*P* = 0.01) and lower maximum spinal cord doses as well (*P* = 0.04) (Table 3). In comparison to DP-IMRT, non-coplanar 3DCRT was found to reduce dose to the left kidney V18 (*P* = 0.006). DP-IMRT was superior to a non-coplanar field arrangement in delivering lower mean liver dose (*P* = 0.001), small bowel V45 (*P* = 0.0002), and stomach V45 (*P* = 0.007). There was no statistically significant difference between the two approaches in terms of right kidney or spinal cord dosimetry.  Figure 3 shows dose distributions for a representative DP-IMRT plan.

## 4. Discussion

The treatment of LAPC is an area of active investigation. Patients are often treated with induction chemotherapy followed by concurrent chemoradiation, although the role of CRT has been questioned [4]. The importance of systemic therapy is suggested by the fact that a large portion of recurrence and treatment failures are a result of metastatic progression of the disease, implying the existence of micrometastases at the time of diagnosis [6, 7]. However, despite formidable research efforts, LAPC continues to present a therapeutic challenge. Patients undergoing these treatment protocols have also been reported to experience various, and sometimes significant, adverse effects, underscoring quality of life concerns.

In the current paper, we investigated the role DP-IMRT may serve in the treatment of LAPC with a focus on improved local control. The OS in our study was approximately 11.6 months, consistent with the published literature, including the RTOG 98-12 and the E4201, which showed overall survivals of 11.2 and 11.1 months, respectively [9, 14]. PFS in our study was found to be 7.2 months, with LRC of 90%, an improvement on several studies reporting 50–78% [6, 15, 16]. Despite the reduction in morbidity that we observed with DP-IMRT, overall survival was similar to published reports and not improved by dose escalation as was found in a recent study in which 24% of patients underwent resection following radiation [17]. The lack of survival benefit in our study may have been the result of small cohort size, advanced disease prior to therapy (precluding radiation delivery), and radiation doses lower than that used by Ben-Josef et al. [17]. Additionally, the large fraction of metastatic recurrence both among our patients and in other reported studies suggests both aggressive biology with underlying micrometastatic disease early in the course of disease and relatively ineffective systemic therapy. Our finding that postradiation CA19-9 strongly correlates with OS supports this notion of aggressive biology. Hence, we are in need of methods of earlier detection of pancreatic cancer and improved chemotherapeutics to improve patient longevity.

The toxicities experienced by the patients were predominantly grades 1 and 2 with one patient experiencing grade 3 nausea and anorexia and one patient exhibiting grade 3 anorexia. Patients in our cohort did not experience any grade 4 adverse events. This is in contrast to published studies utilizing gemcitabine or 5-FU combined with 3D-CRT, in which higher incidences of grade 3 or greater toxicity were observed. Up to 79% grade 3 and 4 adverse events were reported in the Eastern Cooperative Oncology Group (ECOG) trial E4201 which involved 50.4 Gy given concurrently with gemcitabine [9, 12]. This improvement in morbidity may be explained in part by the apparent reduction in radiation dose to OARs we detected after analyzing 10 DP-IMRT plans and comparing to 3D-CRT, with significantly lower doses delivered to the stomach (*P* = 0.007), small bowel (*P* = 0.0002), and liver (*P* = 0.001). It should be noted however that while gemcitabine concurrent with radiation was given at a higher dose in the ECOG trial (600 mg/m^2^/wk versus 400 mg/m^2^/wk in our study), our study incorporated induction gemcitabine of 1000 mg/m^2^ while the ECOG study administered this dose of gemcitabine after radiation. Taken together, these results suggest that DP-IMRT for the treatment pancreatic cancer may decrease morbidity when compared to 3D-CRT, as published reports have suggested in the context of other malignancies [10, 11].

The limitations of the current study are the modest sample size, its retrospective nature, and short follow-up time. Also, while care was taken to ask each patient a standard set of questions regarding adverse events, post hoc analysis inevitably increases the likelihood of underestimating low-grade toxicities. However, the more serious nature of grade 3 and 4 adverse events minimizes the possibility that such levels of toxicity would have been undetected. In addition, the patients in this study received treatments designed and administered by a single institution, minimizing the possibility of institution-based differences.

The present study is novel in its reporting on the treatment of LAPC with DP-IMRT. As a sizable minority of patients die from locoregional progression, the high doses delivered to gross tumor using this technique has the potential to offer improved local control rates leading to OS. DP-IMRT may minimize adverse events by allowing increased sparing of uninvolved tissue. Prospective studies are required to further establish the maximum tolerated dose and degree of LRC offered by DP-IMRT. The feasibility of such future work is suggested by tolerability of DP-IMRT among the patients receiving dose escalation to 54 Gy compared to those receiving 50.4 Gy. Further research in this area can conceivably institute a new treatment regimen with significantly improved local control afforded by the delivery of radiation levels not possible with traditional methods.

In conclusion, DP-IMRT provides a novel approach to the treatment of pancreatic cancer that appears to provide improved local control over conventional methods and results in fewer adverse events, likely due to lower doses of radiation delivered to OARs. Postradiation CA19-9 levels seem predictive of OS in this group of pancreatic cancer patients. Prospective studies are required to compare DP-IMRT to other radiation delivery modalities such as proton therapy or GTV-only irradiation and to investigate the therapeutic advantage of further dose escalation with DP-IMRT both in terms of LRC and improving resection rates.

## Figures and Tables

**Table 1:** Patient demographics and treatment regimens.

Characteristic	*n* (%)
Gender	
Male	14 (54)
Female	12 (46)
Age (years)	
Median (range)	66 (40–87)
T stage	
T2	1 (4)
T3	12 (46)
T4	13 (50)
N stage	
N0	10 (38)
N1	10 (38)
Unknown	6 (23)
Induction chemotherapy	
Gemcitabine	22 (85)
Gemcitabine-paclitaxel-capecitabine (GTX)	1 (4)
5-Fluorouracil-leucovorin-irinotecan-oxaliplatin (FOLFIRINOX)	2 (8)
No induction chemotherapy	1 (4)
Concurrent chemotherapy	
Gemcitabine	7 (35)
5-FU	12 (60)
Both	1 (5)

**Table 2:** Adverse events for patients who received DP-IMRT (*n* = 20).

Toxicity		*n* (%)		
	Grade 0	Grade 1	Grade 2	Grade 3
Diarrhea	12 (60)	4 (20)	4 (20)	
Nausea	4 (20)	9 (45)	6 (30)	1 (5)
Vomiting	11 (55)	6 (30)	3 (15)	
Anorexia	8 (40)	2 (10)	8 (40)	2 (10)
Abdominal pain	13 (65)	6 (30)	1 (5)	
Fatigue	7 (35)	9 (45)	4 (20)	
Weight loss	13 (65)	6 (30)	1 (5)	
Anemia^†^		9 (45)	9 (45)	1 (5)
Gastrointestinal hemorrhage	18 (90)			2 (10)

DP-IMRT: dose painted.

^†^Hematologic toxicity for one patient who was lost to followup could not be determined.

**Table 3:** Radiation delivery to OARs.

Organ	Mean IMRT value	Mean coplanar 3D-CRT value	Mean noncoplanar 3D-CRT value	*P* value (coplanar versus noncoplanar 3D-CRT)	*P* value (IMRT versus noncoplanar 3D-CRT)
Right kidney V12 (%)	75.5	75.6	68.9	**0.03**	0.3
R. kidney V18 (%)	45.5	62.0	58.4	0.6	0.1
Left kidney V12 (%)	69.0	48.3	41.0	0.1	**0.001**
Left kidney V18 (%)	43.2	35.2	26.4	**0.04**	**0.006**
Mean liver dose (Gy)	16.5	20.0	19.5	0.1	**0.001**
Small bowel V45 (%)	7.1	12.1	12.8	**0.01**	**0.0002**
Stomach V45 (%)	8.7	16.6	15.8	0.2	**0.007**
Max spinal cord dose	35.0	32.0	35.5	**0.04**	0.8

OAR: organ at risk; IMRT: intensity-modulated radiation Therapy; 3D-CRT: three-dimensional conformal radiation therapy; V_*x*_: volume of an organ receiving *X* Gy.
